# Supplementary material for: Sub-continental-scale carbon stocks of individual trees in African drylands
Source: Nature. 2023 Mar 1;615(7950):80–6. doi: 10.1038/s41586-022-05653-6 (PMC9977681; doi:10.1038/s41586-022-05653-6)
Supplement: Supplementary file 1 — This file contains Supplementary Fig. 1a–j and Supplementary Data figure information [file 41586_2022_5653_MOESM1_ESM.pdf]

nature portfolio

https://doi.org/10.1038/s41586-022-05653-6

Supplementary information

Sub-continental-scale carbon stocks of individual trees in African drylands

In the format provided by the authors and unedited

Supplemental Information Figures

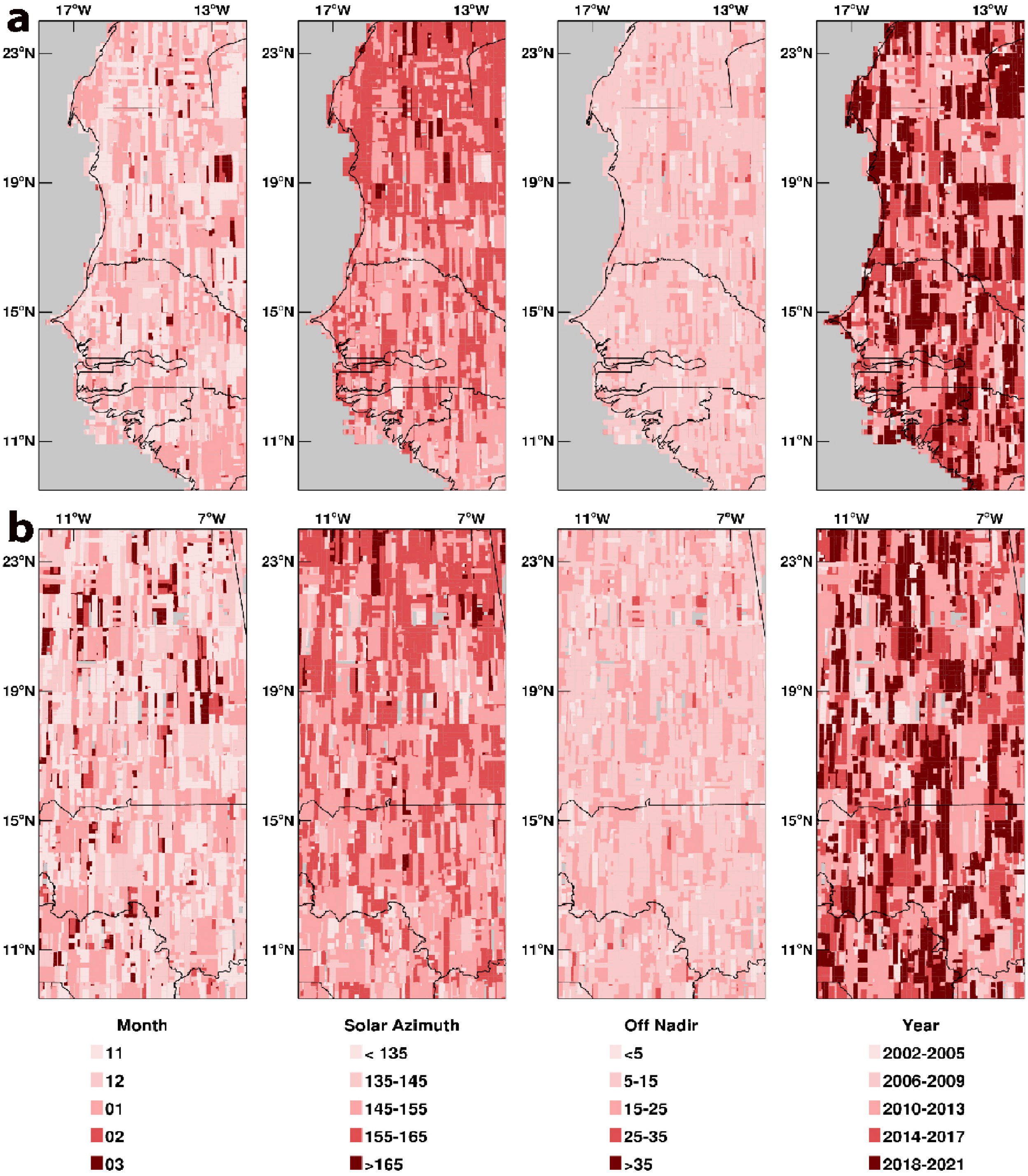

Supplemental Information Fig. 1a & 1b | UTM Zones 28 & 29 satellite data particulars. a, For UTM Zone 28 there were 42,388 candidate Maxar images between 9.5° to 24° N latitude for this UTM Zone segment and 8,026 were selected for processing. **b,** For UTM Zone 29 there were 29,244 candidate Maxar images between 9.5° to 24° N latitude for this UTM Zone segment and 9,883 images were selected for processing. The distribution of the data with respect to month, solar azimuth, off-nadir angle, and year of acquisition are given for each UTM Zone segment.

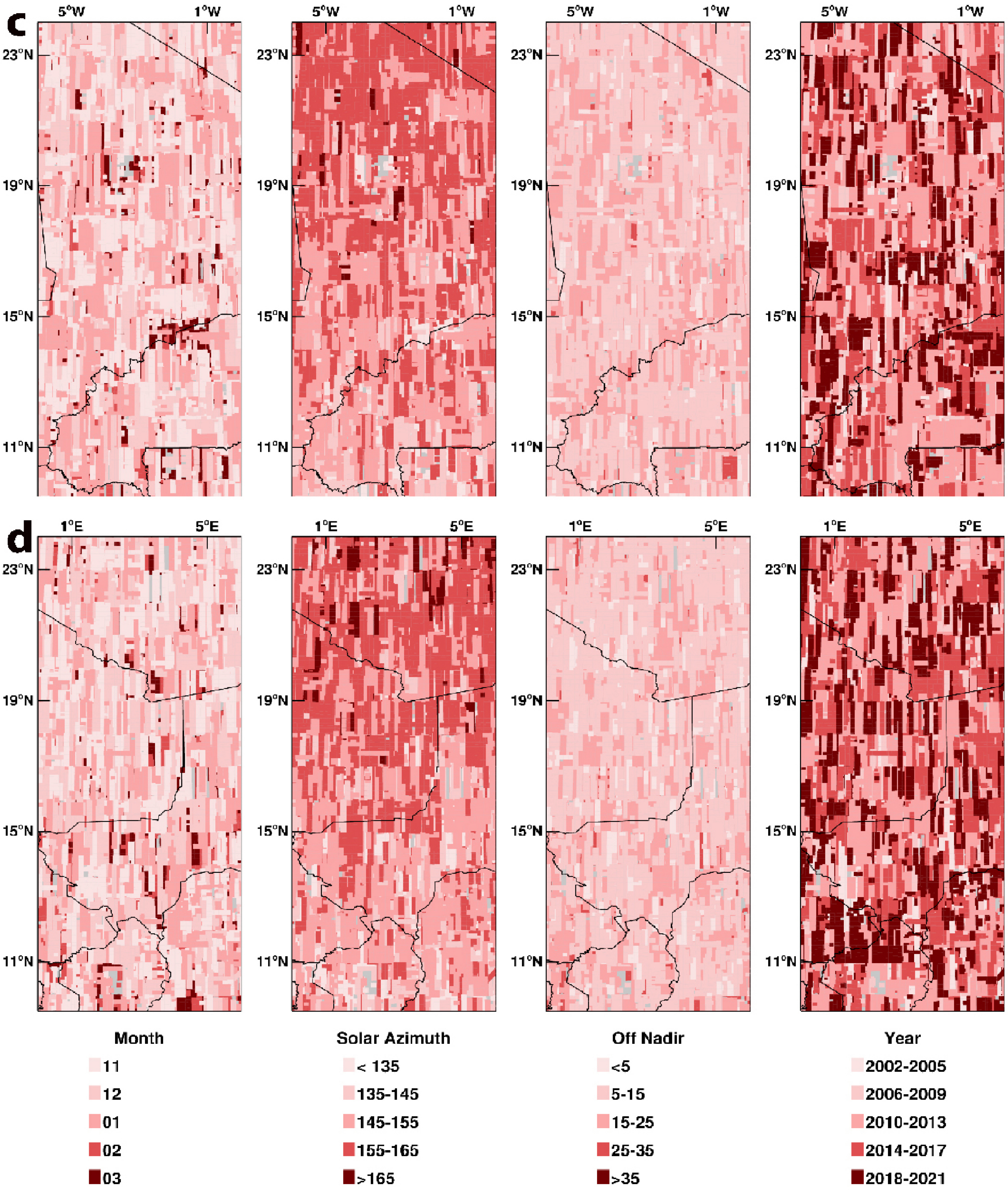

Supplemental Information Fig. 1c & 1d | UTM Zones 30 & 31 satellite data particulars. **c**, For UTM Zone 30 there were 32,671 candidate Maxar images between 9.5° to 24° N latitude for this UTM Zone segment and 10,155 were selected for processing. **d**, For UTM Zone 31 there were 34,611 candidate Maxar images between 9.5° to 24° N latitude for this UTM Zone segment and 10,158 were selected for processing. The distribution of the data with respect to month, solar azimuth, off-nadir angle, and year of acquisition are given for each UTM Zone segment.

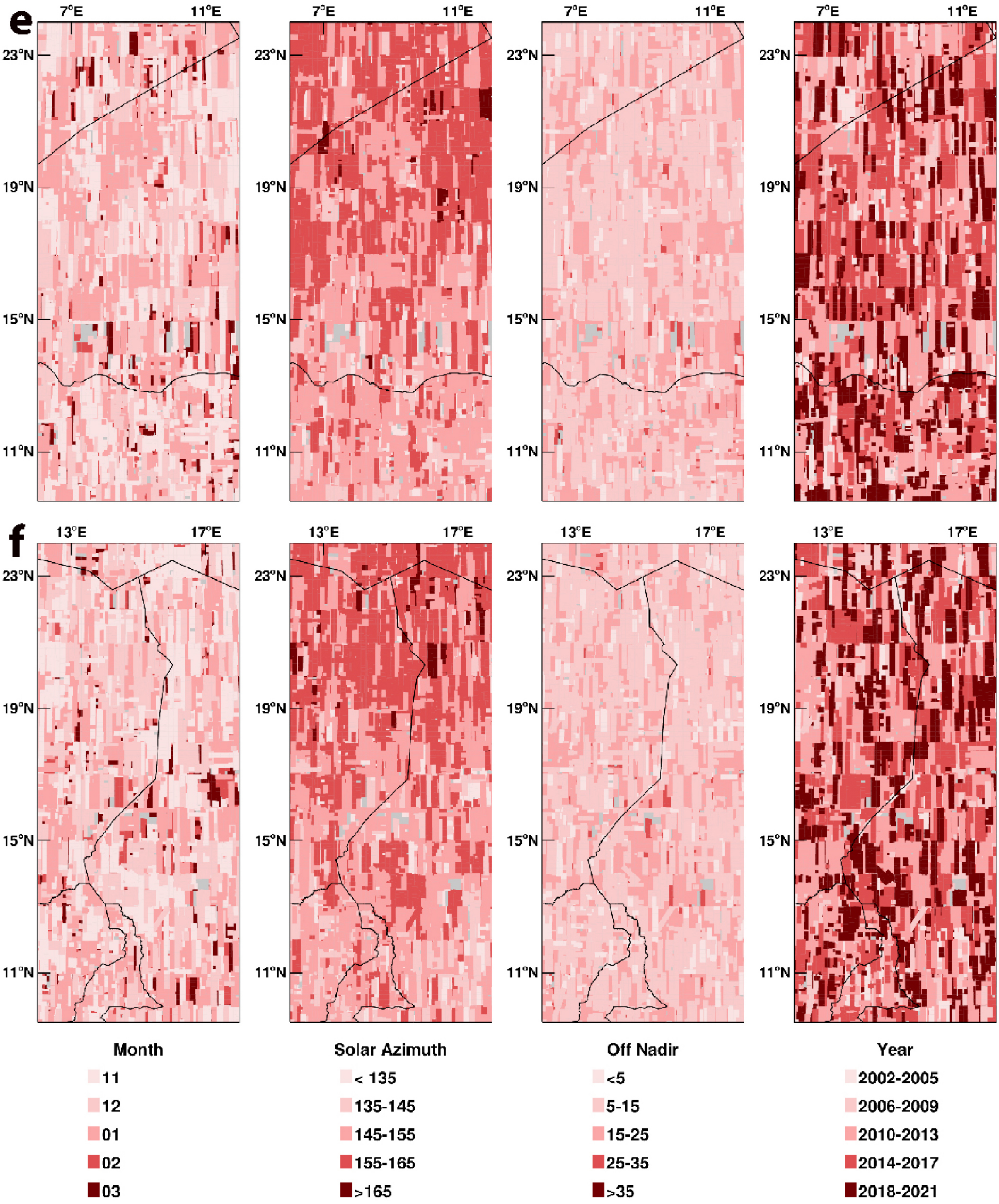

Supplemental Information Fig. 1e & 1f | UTM Zones 32 & 33 satellite data particulars. e, For UTM Zone 32 there were 32,210 candidate Maxar images between 9.5° to 24° N latitude for this UTM Zone segment and 9,971 were selected for processing. **f,** For UTM Zone 33 there were 34,918 candidate Maxar images between 9.5° to 24° N latitude for this UTM Zone segment and 10,097 were selected for processing. The distribution of the data with respect to month, solar azimuth, off-nadir angle, and year of acquisition are given for each UTM Zone segment.

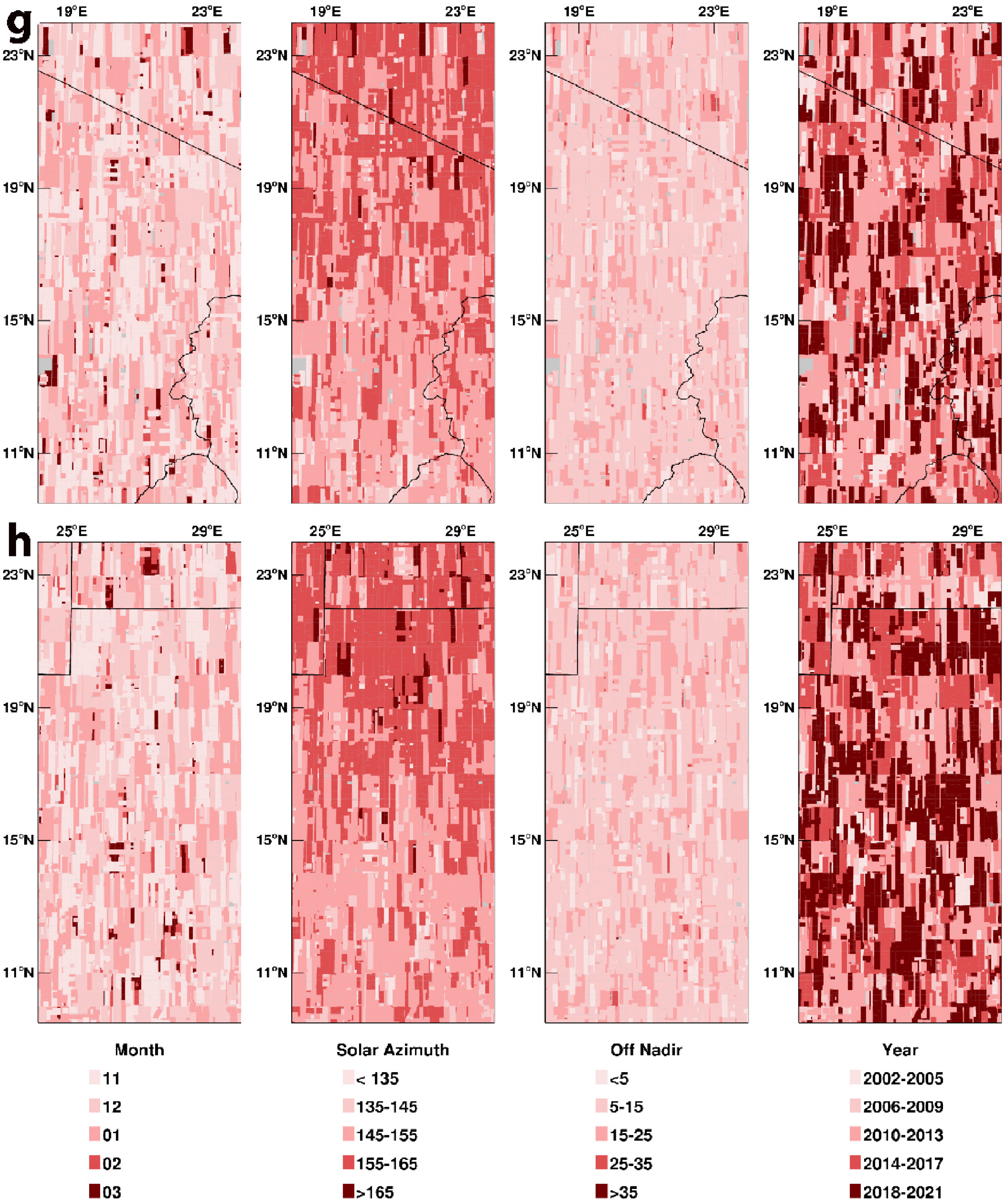

Supplemental Information Fig. 1g & 1h | UTM Zones 34 & 35 satellite data particulars. g, For UTM Zone 34 there were 31,555 candidate Maxar images between 9.5° to 24° N latitude for this UTM Zone segment and 10,144 were selected for processing. **h**, For UTM Zone 35 there were 36,663 candidate Maxar images between 9.5° to 24° N latitude for this UTM Zone segment and 9,975 were selected for processing. The distribution of the data with respect to month, solar azimuth, off-nadir angle, and year of acquisition are given for each UTM Zone segment.

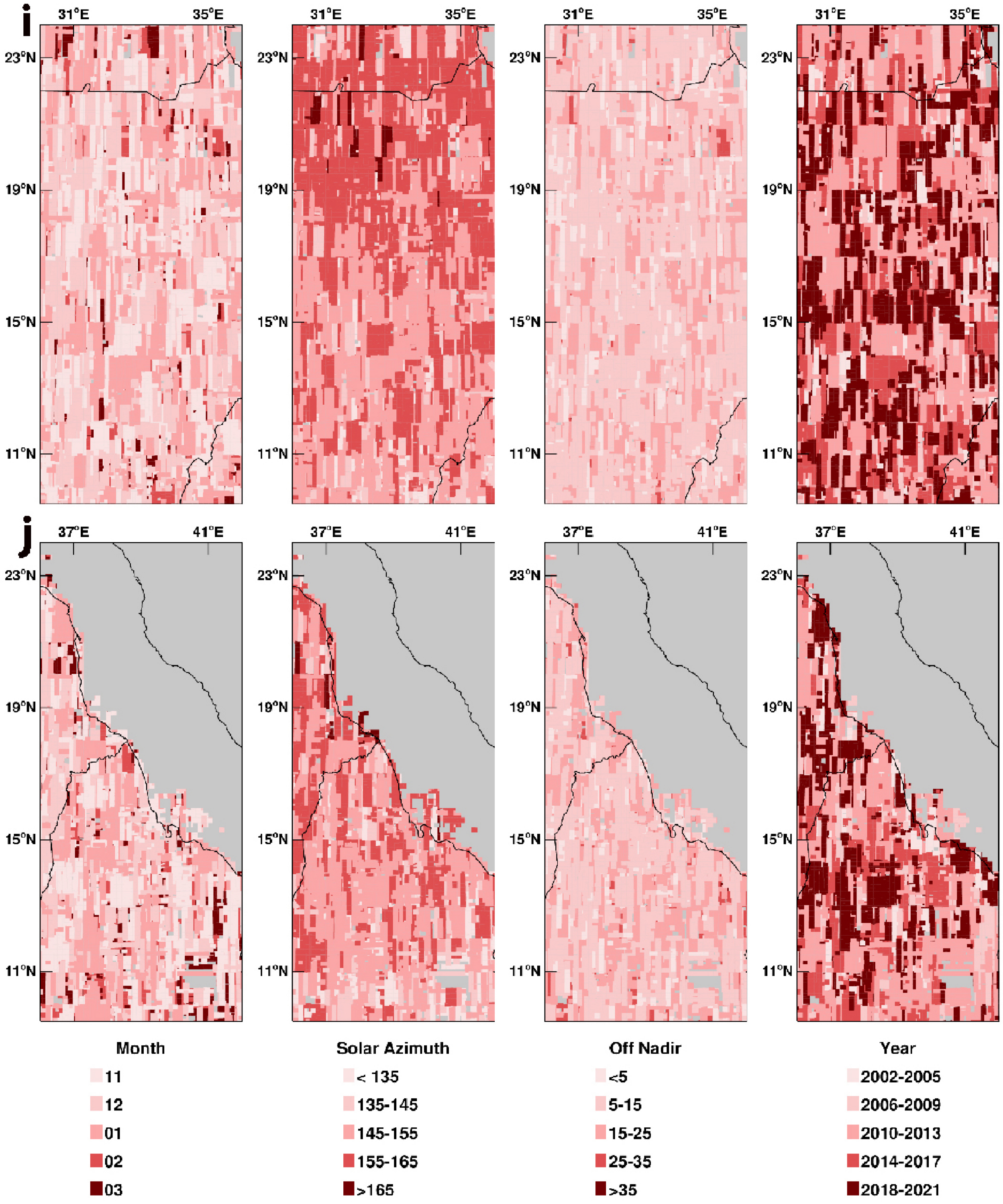

Supplemental Information Fig. 1i & 1j | UTM Zones 36 & 37 satellite data particulars. i, For UTM Zone 36 there were 36,934 candidate Maxar images between 9.5° to 24° N latitude for this UTM Zone segment and 10,156 were selected for processing. **j**, For UTM Zone 37 there were 15,329 candidate Maxar images between 9.5° to 24° N latitude for this UTM Zone segment and 5,937 were selected for processing. The distribution of the data with respect to month, solar azimuth, off-nadir angle, and year of acquisition are given for each UTM Zone segment.

Supplementary Data

Supplementary Data Figure Information. Supplementary Information Fig. 1 provides information by UTM Zone segment from 9.5^{o} N to 24° N latitude and describe aspects of the satellite data used in our study for portions of UTM Zones 28 to 37. These stretch from the Atlantic Ocean to the Red Sea across Africa north of the equator and south of the Central Sahara Desert (fig. 1 in our paper). We describe the number of candidate satellite images considered for processing, the number of candidate images that were selected for processing, and details of the selected images processed: month of year; solar azimuth; off-nadir angle; and year of acquisition. A total of 94,502 satellite images were selected for processing for our 9,685,324 km^2 study area by UTM Zone segment. See also Extended Data Fig. 8 and Extended Data Table 1a in our paper.
